# Pain curricula across healthcare professions undergraduate degrees: a cross-sectional study in Catalonia, Spain

**DOI:** 10.1186/s12909-019-1741-5

**Published:** 2019-08-13

**Authors:** Jordi Miró, Elena Castarlenas, Ester Solé, Lorena Martí, Isabel Salvat, Francisco Reinoso-Barbero

**Affiliations:** 1 Universitat Rovira i Virgili, Unit for the Study and Treatment of Pain-ALGOS, Chair in Pediatric Pain URV-FG, Research Center in Beahvior Assessment and Measurement, Department of Psychology, Tarragona, Catalonia Spain; 2 Institut d’Investigació Sanitària Pere Virgili, Tarragona, Spain; 3 School of Medicine and Health Sciences, Department of Physiotherapy, Universitat Rovira i Virgili, Tarragona, Spain; 4 Servicio de Anestesiología-Reanimación Infantil, Unidad de Dolor Infantil, Hospital Universitario La Paz, Madrid, Spain; 5 Departament de Psicologia, Universitat Rovira i Virgili, Carretera de Valls, s/n, 43007 Tarragona, Spain

**Keywords:** Pain education, Curriculum, Undergraduate training, Teaching methods, Health sciences

## Abstract

**Background:**

Pain management is a challenge and effective treatment requires professionals to collaborate if they are to address the needs of patients with pain. Comprehensive education and training is key to helping skilled professionals provide the best pain care possible. The objective of this work was to study the content of the pain education provided to undergraduates in healthcare and veterinary programs in Spain.

**Methods:**

A survey was developed on the basis of previous surveys that had been used in the field. The final version included 31 questions about different issues on pain education, including, type of subject, number of pain mandatory/elective hours, and specific content covered. The survey was sent to all course leaders for all subjects on the undergraduate programs in Dentistry, Human Nutrition and Dietetics, Medicine, Nursing, Occupational Therapy, Pharmacy, Physiotherapy, Podiatry, Psychology, and Veterinary Science, in Catalonia, Spain. The survey was conducted from January to June, 2018. Students’ t-test were used to study mean differences in responses.

**Results:**

A total of 550 course leaders from all healthcare undergraduate programs in Catalan universities took part. There were considerable differences in the number of pain-related hours among disciplines: Nursing reported the highest number of hours, and Psychology the lowest. The area least covered by all the disciplines was the “Management of pain”, and particularly the content related to the most vulnerable members of society (i.e., youths, the elderly and special populations). No interprofessional educational program on pain was identified.

**Conclusions:**

Pain is not such a large component of the undergraduate healthcare curriculum in Spain as could be expected given the extent of pain and its impact. Curricula need to be changed so that the problems all stakeholders have with pain care can be addressed.

## Introduction

Despite advances in the treatment of individuals with chronic pain [1], patients do not always receive the treatment they need, as access to treatment is still a major challenge for many [2]. For example, less than 25% of the world’s population has access to effective medication to alleviate moderate-to-severe pain [3].

Knowledge deficits in pain management among health care practitioners have been claimed to be one of the most important barriers to improving pain relief and care worldwide [4, 5]. Research has shown that future clinicians are left without the training and information they require to help individuals with pain [6]. For example, a recent study on the treatment of young people with chronic pain in Spain reported that 86% of the pediatricians and 80% of the primary-care practitioners participating indicated that they had been given little if any training in the management of pain during their undergraduate education [7].

Studies on pain curricula have shown that the number of teaching hours devoted to pain management is inadequate. For example, the seminal study conducted by Watt-Watson and colleagues [8] compared the pain content in pre-licensure curricula in the health and veterinary sciences in ten major Canadian universities. The study revealed that the number of teaching hours varied between universities, ranging from 0 to 109 across all years, with an average of between 13 and 41 h. On average, veterinary students received five times more hours of pain teaching content than medical students. Two other studies have also compared pain education programs in various health professions: one was conducted in the United Kingdom [9], and the other in Norway [10], and the results of both show the very few hours devoted to pain. Although these three studies reported on pain education programs in several health care disciplines, they left out some that have important implications. For example, only Leegaard and colleagues included Psychology, a discipline that is key to the treatment of individuals with pain, particularly chronic pain, and they received limited information from just one participant in the study.

In an attempt to improve professional competency, organizations such as the International Association for the Study of Pain and the European Federation of IASP Chapters have developed pain curricula to promote training and improve pain education among professionals. These educational guidelines are now asked to reflect the multidimensionality of the pain experience, as well as the need for an interdisciplinary approach to the treatment of individuals with (chronic) pain [11], but the extent to which this is being implemented in the training of future health care professionals is not clear.

Therefore, the aim of this study was to describe the content of pain curricula in all undergraduate healthcare programs in public and private universities in Spain, and identify how much interprofessional training is provided.

## Methods

### Procedure

In Spain, undergraduate studies are essentially the same among universities, as the courses are mostly mandatory and the contents specifically identified in the Official Bulletin of the Nation (*Boletín Oficial del Estado*). Thus, in this study, as a way to address an otherwise unmanageable amount of information, we decided to limit our request for information to the universities from one of the regions in Spain: Catalonia. Therefore, this cross-sectional study included all the undergraduate programs in healthcare at the eleven public and private universities in Catalonia: dentistry, medicine, nursing, physiotherapy, nutrition, psychology, podiatry, occupational therapy, and pharmacy. As in previous similar studies, veterinary science was also included for purposes of comparison (e.g., 8,9). A total of 1564 course leaders were identified by reviewing the curricula and the teaching guidelines available on the website of each university, and they were asked to participate by responding to an online survey. If a particular course leader was in charge of more than one subject, they were asked to report on each subject.

This survey was implemented using the REDCap® program (www.projectredcap.org). Potential participants were sent an email explaining the study and requesting their participation; two reminders were sent encouraging those that did not respond to participate.

### Measure

For the purposes of this study we developed a specific survey on the basis of previous surveys that have been used in the field [8]. Participants were asked if the subject for which they were responsible included any pain content. If it did, they also had to provide the name of the subject, the type of subject according to the curriculum (i.e., mandatory or elective) and the total number of hours it involved. In order to identify what type of pain content was taught, we used the IASP curriculum guidelines as a template and structured the survey in the four general areas they describe: [1] Multidimensional Nature of Pain; [2] Pain Assessment and Measurement; [3] Management of Pain; and [4] Clinical Conditions. This fourth area focuses on the role of the professional in applying the knowledge covered in the previous three domains in a variety of populations (e.g., older adults, children). The survey contained a final section that had to be answered by all participants, regardless of whether their subjects included pain content or not. This section asked three questions on [1] the importance of pain education for a healthcare practitioner; [2] the adequacy of the pain education provided in the curriculum; and [3] the need for additional time and resources for pain education in their curriculum. These questions were to be responded using a 5-point rating scale (1 = Totally disagree to 5 = Totally agree). To test the clarity and appropriateness of the questions, the survey was pilot tested in a small group of pain experts (*n* = 5), who suggested no changes. So, the survey was sent to potential participants as it was.

### Data analysis

We first computed descriptive analyses for the demographic variables to describe the sample of participants. Second, we examined the structure and the content of the curricula. The percentages of courses with pain content were obtained (for the total sample and each discipline/study). We also computed the averages of pain content hours for each content category according to the IASP curriculum guidelines. Third, we compiled and categorized the responses to the questions about the importance and adequacy of pain education and the resources used. Finally, we examined if there were significant mean differences in the responses to the three questions given by the course leaders who included pain content in their subjects and those who did not by conducting a Students’ t-test for each question. These tests were performed for the total sample and for each discipline/study.

## Results

### Sample characteristics

Course leaders from all healthcare (i.e., Dentistry, Human Nutrition and Dietetics, Medicine, Nursing, Occupational Therapy, Pharmacy, Physiotherapy, Podiatry, and Psychology) and Veterinary Science undergraduate programs of the 11 private and public Catalan universities participated in the survey. A total of 550 course leaders participated (35% of those invited). Table 1 shows response rates for programs/disciplines. The response rate ranged from 31% in Pharmacy and Physiotherapy to 55% in Dentistry.

**Table 1:** Response rate and average total hours of pain content by program/discipline

	Total participation		Courses with pain content			
Disciplines	n	%	n	Courses	Total hours, mean (SD)	Range
Dentistry	39	55	28	42	115 (47)	94–137
Human Nutrition and Dietetics	35	39	11	19	9 (8)	3–22
Medicine	136	37	70	95	70 (47)	10–127
Nursing	117	47	71	108	87 (65)	21–230
Occupational Therapy	15	26	3	7	13 (9)	7–20
Pharmacy	20	31	5	6	7 (5)	4–11
Physiotherapy	98	31	65	100	97 (34)	34–129
Podiatry	16	37	9	12	88 (21)	73–103
Psychology	102	41	22	24	26 (29)	2–75
Veterinary Science	30	53	9	13	103 (0)	103-103^a^

Note: The number of subjects is higher than the number of participants because one leader can lead more than one subject. The variable “Total hours, mean” is the mean number of hours for all the universities for that specific program/discipline. Range includes the lowest and the highest total number of hours per discipline for all the universities included in the study

^a^Just one university offers Veterinary Science as an undergraduate program

### Structure of pain curricula

**Fig. 1:**
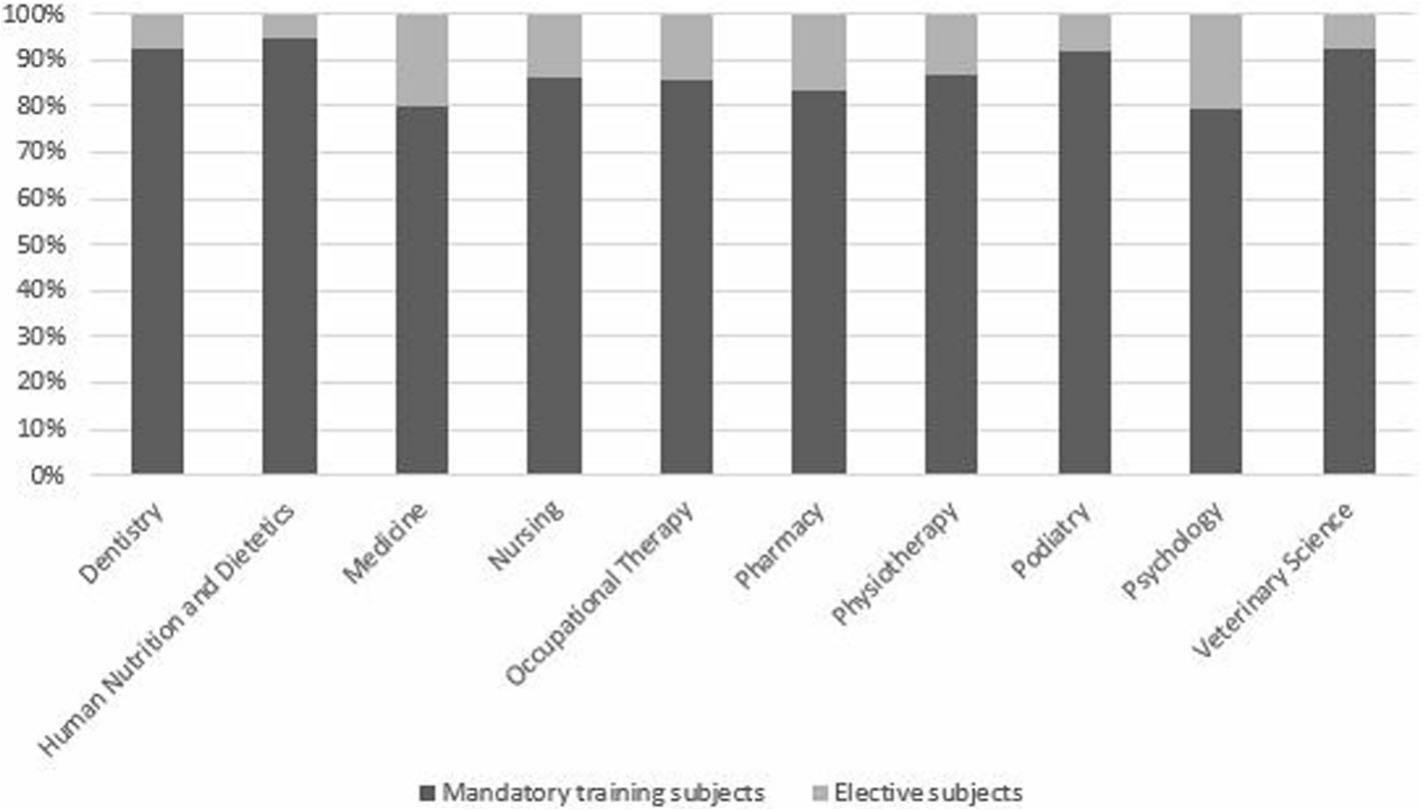
Types of subject with pain content by discipline

A total of 244 participants (44%) reported that the subjects they teach have pain content. The course leaders reported information on 367 subjects. Of these, 84% were mandatory and 16% were elective. Figure 1 illustrates the percentages of each type of subject by discipline.

The total average of hours of pain content varied among programs (see Table 1). Dentistry and Veterinary Science reported the highest mean number of hours: 115 and 103 h, respectively, out of 3000 h per degree (3.8 and 3.4%, respectively). Nursing showed the highest upper range number of hours (230 out of 2400 h per degree; or 9.6%), and three disciplines reported less than 5 h in the lowest range: Psychology (2 h out of 2400 h per degree; or 0.1%), Nutrition (3 h out of 2400 h per degree; or 0.1%), and Pharmacy (4 h out of 3000 h per degree; or 0.1%).

### Type of pain content

Table 2 illustrates the percentages of each general area and specific pain content for all programs/disciplines.

**Table 2:** Specific pain content that participants teach in subjects

	Dentistry	Human Nutrition and Dietetic	Medicine	Nursing	Occupational Therapy	Pharmacy	Physiotherapy	Podiatry	Psychology
Multidimensional nature of pain (13.09)	(23.9)	(7.2)	(16)	(18.7)	(4.5)	(6.7)	(17.9)	(17.7)	(5.2)
Epidemiology of pain	4 (10.3)	1 (2.9)	10 (7.4)	11 (9.4)	0 (0)	0 (0)	7 (7.1)	1 (6.3)	1 (1)
Pain theories	5 (12.8)	1 (2.9)	7 (5.1)	8 (6.8)	0 (0)	0 (0)	8 (8.2)	2 (12.5)	5 (4.9)
Biological mechanisms of pain	11 (28.2)	5 (14.3)	37 (27.2)	20 (17.1)	1 (6.7)	3 (15)	23 (23.5)	3 (18.8)	7 (6.9)
Ethical standards and guidelines related to management of pain	5 (12.8)	1 (2.9)	9 (6.6)	18 (15.4)	1 (6.7)	0 (0)	7 (7.1)	1 (6.3)	0 (0)
Definition of pain and pain terms	19 (48.7)	6 (17.1)	43 (31.6)	42 (35.9)	1 (6.7)	4 (20)	37 (37.8)	6 (37.5)	10 (9.8)
Biological, psychological and social factors influencing the perception of pain	12 (30.8)	1 (2.9)	25 (18.4)	32 (27.4)	1 (6.7)	1 (5)	23 (23.5)	4 (25)	9 (8.8)
Pain assessment and measurement (13.56)	(23.1)	(7.9)	(16.3)	(23.5)	(6.7)	(1.2)	(18.6)	(20.3)	(4.4)
Interprofessional and multiprofessional collaboration	6 (15.4)	3 (8.6)	20 (14.7)	23 (19.7)	1 (6.7)	0 (0)	13 (13.3)	4 (25)	2 (2)
Pain assessment	15 (38.5)	4 (11.4)	37 (27.2)	39 (33.3)	1 (6.7)	1 (5)	27 (27.6)	4 (25)	5 (4.9)
Pain impact on quality of life	11 (28.2)	3 (8.6)	23 (16.9)	30 (25.6)	2 (13.3)	0 (0)	23 (23.5)	3 (18.8)	8 (7.8)
Evaluation of outcomes	4 (10.3)	1 (2.9)	9 (6.6)	17 (15.5)	0 (0)	0 (0)	10 (10.2)	2 (12.5)	3 (2.9)
Management of pain (10.28)	(17.5)	(6.7)	(13.3)	(17.8)	(3.3)	(4.2)	(13.9)	(12.5)	(3.3)
Pharmacological methods	13 (33.3)	6 (17.1)	34 (25)	36 (30.8)	0 (0)	4 (20)	10 (10.2)	4 (25)	0 (0)
Non-pharmacological approaches	9 (23.1)	3 (8.6)	24 (17.6)	33 (28.2)	0 (0)	0 (0)	24 (24.5)	2 (12.5)	7 (6.9)
Psychological approaches	8 (20.5)	2 (5.7)	20 (14.7)	23 (19.7)	2 (13.3)	1 (5)	12 (12.2)	1 (6.3)	12 (11.8)
Rehabilitation approaches	4 (10.3)	1 (2.9)	12 (8.8)	5 (4.3)	0 (0)	0 (0)	22 (22.4)	3 (18.8)	0 (0)
Surgical approaches	3 (7.7)	1 (2.9)	9 (6.6)	12 (10.3)	0 (0)	1 (0)	2 (2)	0 (0)	0 (0)
Other non-pharmacological	4 (10.3)	1 (2.9)	10 (7.4)	16 (13.7)	1 (6.7)	0 (0)	12 (12.2)	2 (12.5)	1 (1)
Clinical conditions (13.62)	(24.5)	(8.6)	(16.5)	(17.8)	(2.9)	(7.8)	(19)	(22.3)	(3.2)
Type(s) of pain (neuropathic pain, nociceptive pain)	17 (43.6)	8 (22.9)	49 (36)	40 (34.2)	1 (6.7)	3 (15)	38 (38.8)	8 (50)	6 (5.9)
Distinction between commonly used pain terms in clinical practice (e.g. allodynia, analgesia, dysesthesia, hyperalgesia)	9 (23.1)	2 (5.7)	22 (16.2)	14 (12)	0 (0)	2 (10)	20 (20.4)	4 (25)	0 (0)
Pain in older adults	6 (15.4)	0 (0)	6 (4.4)	14 (12)	0 (0)	0 (0)	3 (3.1)	1 (6.3)	1 (1)
Pain in special population (e.g., pain in people with psychiatric disorder or in individuals with substance abuse)	2 (5.1)	0 (0)	6 (4.4)	14 (12)	0 (0)	0 (0)	4 (4.1)	1 (6.3)	5 (4.9)
Distinction between acute, recurrent, incident, and or persistent pain	19 (48.7)	8 (22.9)	48 (35.3)	43 (36.8)	1 (6.7)	3 (15)	38 (38.8)	7 (43.8)	8 (7.8)
Pain in infants, children and adolescents	3 (7.7)	0 (0)	5 (3.7)	8 (6.8)	1 (6.7)	0 (0)	2 (2)	2 (12.5)	1 (1)
Specific pain problems (e.g., back pain, headache)	11 (28.2)	3 (8.6)	21 (15.4)	13 (11.1)	0 (0)	3 (15)	25 (25.5)	2 (12.5)	2 (2)

Note: Data presented for subcategories as percentages (%) or number of respondents and percentages [n (%)], and data presented for general categories as percentages (%)

Content from the area “Clinical conditions” occurred most frequently (13.62%) whereas content from “Management of pain” occurred the least frequently (10.28%). The content that was least dealt with in each area was “Pain in infants, children and adolescents” (4%), “Pain in older adults” (5%), “Pain in special populations” (5%), and “Pain theories” (5%). The content that was most dealt with was the following: “Pain assessment” (21%), “Biological, psychological and social factors influencing the perception of pain” (17%), “Non-pharmacological approaches” (16%) and “Interprofessional and multidisciplinary collaboration” (12%).

### Course leaders’ opinion about pain content in the curricula

Most of the participants in the study agreed on the importance of pain education: the lowest score was for course leaders from Psychology [Mean (SD) = 3.81 (0.91)], and the highest for those from Dentistry [Mean (SD) = 4.85 (0.36)].

In general, our participants did not consider pain education to be adequate in its current form. This can be seen in the responses from course leaders in Psychology, Medicine, and Human Nutrition and Dietetics. However, the leaders from Dentistry, Podiatry and Nursing seemed to agree more that pain education is adequate as it is now given.

Finally, course leaders also acknowledged that more resources and time should be invested in pain education during undergraduate training, with consensus being greatest in Physiotherapy and Dentistry, and lowest in Pharmacy, Psychology and Medicine (see Table 3).

**Table 3:** Participants’ level of agreement on opinion questions about pain content in healthcare professionals’ curricula by discipline

	Dentistry	Human Nutrition and Dietetics	Medicine	Nursing	Occupational Therapy	Pharmacy	Physiotherapy	Podiatry	Psychology	Veterinary Science
Importance of pain education in the training of their students, future clinicians	4.85 (0.36)	4.09 (0.89)	4.09 (0.89)	4.81 (0.4)	4.47 (0.52)	4.2 (0.62)	4.77 (0.56)	4.73 (0.59)	3.81 (0.91)	4.52 (0.75)
Adequacy of the pain education provided in their curriculum	3.59 (0.89)	2.91 (0.89)	2.91 (0.89)	3.26 (0.91)	3.00 (1.13)	3.50 (0.69)	3.16 (0.91)	3.27 (0.96)	2.83 (0.85)	3.22 (0.93)
Need for additional time and resources for pain education in their curriculum, importance of pain curricula in undergraduate degrees	4.06 (0.85)	3.53 (0.84)	3.53 (0.84)	4.03 (0.74)	3.87 (0.64)	3.35 (0.67)	4.09 (0.84)	3,93 (0.70)	3.51 (0.82)	3.63 (0.88)

Note: Data is presented as Mean (SD); Range of responses = 1–5

When we compared the opinions of course leaders on these three issues, statistically significant differences emerged between those who included pain content in their subjects and those who did not. Specifically, those who included pain content in their subjects [Mean (SD) = 4.75 (0.54)] were more in agreement that a good training in pain during undergraduate education is key to the students’ future as health-care practitioners than those who did not [Mean (SD) = 4.26 (0.84); (t (487.37) = 7.92, *p* < 0.01). They were also more in agreement that student’s training and education on pain is adequate as it is now provided for their professional future [Mean (SD) = 3.27 (0.96)] than those who did not include pain content in their subjects [Mean (SD) = 3.0 (0.86); (t (427.51) = 3.16, *p* < 0.01). However, course leaders who did not include pain content in their subjects showed a higher level of agreement on the need to invest more resources and time on training undergraduates in pain-related content [Mean (SD) = 4.0 (0.85)] than course leaders who did include pain contents [Mean (SD) = 3.63 (0.83); t (454.35) = 4.83, *p* < 0.01].

## Discussion

This study provides new important information on pain education for healthcare and veterinary professionals in Catalonia, Spain. To the best of our knowledge, it is unique in that it is the only study that has surveyed all undergraduate degrees in the health sciences (i.e., Dentistry, Human Nutrition and Dietetics, Medicine, Nursing, Occupational Therapy, Pharmacy, Physiotherapy, Podiatry, and Psychology), thus making it possible to compare the situation of pain education across the spectrum. Another strength of the study is the implication of a large number of participants (course leaders). Unlike previous studies, we directly asked those in charge of teaching the subjects, whereas previous surveys asked one person, normally the Dean or a representative, to inform about how the students were taught in each program (e.g., [10, 12]) even though it is unlikely that a single person can reliably inform about the content of all courses.

The results show that pain education is still a marginal issue in the undergraduate degrees for health-care professionals, as can be seen by the little time given to the teaching of pain-related issues, particularly pain management.

The total number of hours devoted to pain education varied greatly among programs. The highest totals ranged from 230 h in Nursing to 11 h in Pharmacy, whereas the lowest totals ranged from the 2 h in Psychology to the 103 h in Veterinary Science. These results are similar to those reported in previous studies in Canada [8], Norway [10], and the United Kingdom [9]. For example, the mean number of hours devoted to pain education in Medicine in each of these programs were, respectively: 16, 13, 31.

The highest variance in hours was reported in Medicine (range = 10–127) and Nursing (range = 21–230), compared to Pharmacy (range = 4–11), which showed the greatest homogeneity in responses. Most of the pain-related content was provided in compulsory subjects. It is encouraging to notice the inclusion of content related to the biopsychosocial model in all programs, as this has been identified as key to improving treatment for individuals with chronic pain in particular [14, 15]. However, the data also highlights the need to increase both the breadth and depth of pain content so that healthcare professionals can provide individuals in pain with better treatment.

This study has also shown that there is a great deal of pain-related content in the undergraduate curricula of healthcare and veterinary students, but it is unclear what students are required to do, whether they need merely to memorize content or do a little more. Moreover, this study provides no insight into the effectiveness of the pain content of the undergraduate training of these future professionals. Thus, future studies are needed to address these issues so that the treatment of individuals with pain can be improved.

The data gathered in this survey show that pain education is regarded as important by most course leaders. They also perceive that the training and education provided is not adequate, and that additional resources and time should be invested in undergraduate curricula. These results are in line with those from a recent survey on the treatment of pediatric chronic pain in Spain. In this other study, participants (i.e., primary care practitioners and pediatricians) informed that the training received was insufficient, and that the lack of training was the most important barrier to helping their patients. In fact, they reported that they had essentially learned pain management on the job as [7].

As has been reported in previous studies in the field (e.g., [13]), we did not find a single *interprofessional* educational program (IPE) on pain (i.e., a program in which two or more professions learn with, from, and about each other to improve collaboration and the quality of care; [16], or *intraprofessional* educational program (i.e., a program coordinated between departments), even in Medicine or Nursing which have the longest tradition of pain education, in any of the universities. The *uniprofessional* education of the programs surveyed in this study does not help students learn the competencies required for professional work [17]. Uniprofessional education might result in a fragmented and therefore inadequate understanding of pain, more like a symptom than a disease entity. In addition, it does not prepare students to work in teams, which is essential, particularly when working with populations with chronic pain [18]. Data shows that when students from various professions learn together and interact, they improve their communication skills, enhance future relationships, and improve performance while on the job [5]. Although IPE is difficult to implement, depending on the situation, various scalable alternatives could be used: for example, integrating a module into the curriculum in all the degrees at the same university, as is done at the University of Washington [4] or developing a complete specific pain curricula [19]. Research needs to be done to determine which approach is best for every circumstance.

This study is not exempt from limitations that should be borne in mind when interpreting the results. First, the response rate was low for some programs. Nevertheless, it is very similar to that reported in previous studies (e.g., [10, 13]). Second, the data used was provided by a group of course leaders, all of whom wanted to participate, therefore this might have impacted on their responses in ways that we do not know. However, we have collected information from undergraduate programs from all the public and private universities in Catalonia, Spain, so it is reasonable to expect that the data are representative for Spain. Third, social desirability is also a potential limitation of the study, even though the responses could be confirmed, to some extent, with information that is publicly available, which might have limited this potential threat to validity. Besides, the results are similar to those found in other countries (e.g., [14, 20]).

## Conclusions

Regardless of its limitations, this is the first survey to inform on the pain content from all undergraduate health-care programs in Catalonia, Spain. It identifies some concerns: [1] the depth and breadth of the pain content taught is not sufficient, particularly in the area of pain management and in relation to the most vulnerable populations (e.g., the young); [2] the investment in pain education in terms of resources and time during undergraduate training is limited; and [3] the most widely used educational model is the uniprofessional one, which is now obsolete. However, it also raises important positive issues, the most significant of which is that the biopsychosocial model is taught in all programs, which was not the case not so long ago. Therefore, the findings seem to indicate that there is progress in the field, but that pain education (linked to the interprofessional model) needs to be better organized and delivered to correct the shortcomings in the provision of pain care for all those in need.

## Data Availability

The dataset used and analyzed during the current study are available from the corresponding author on reasonable request.

## Abbreviations

IASP: International Association for the Study of Pain
IPE: Interprofessional Educational
